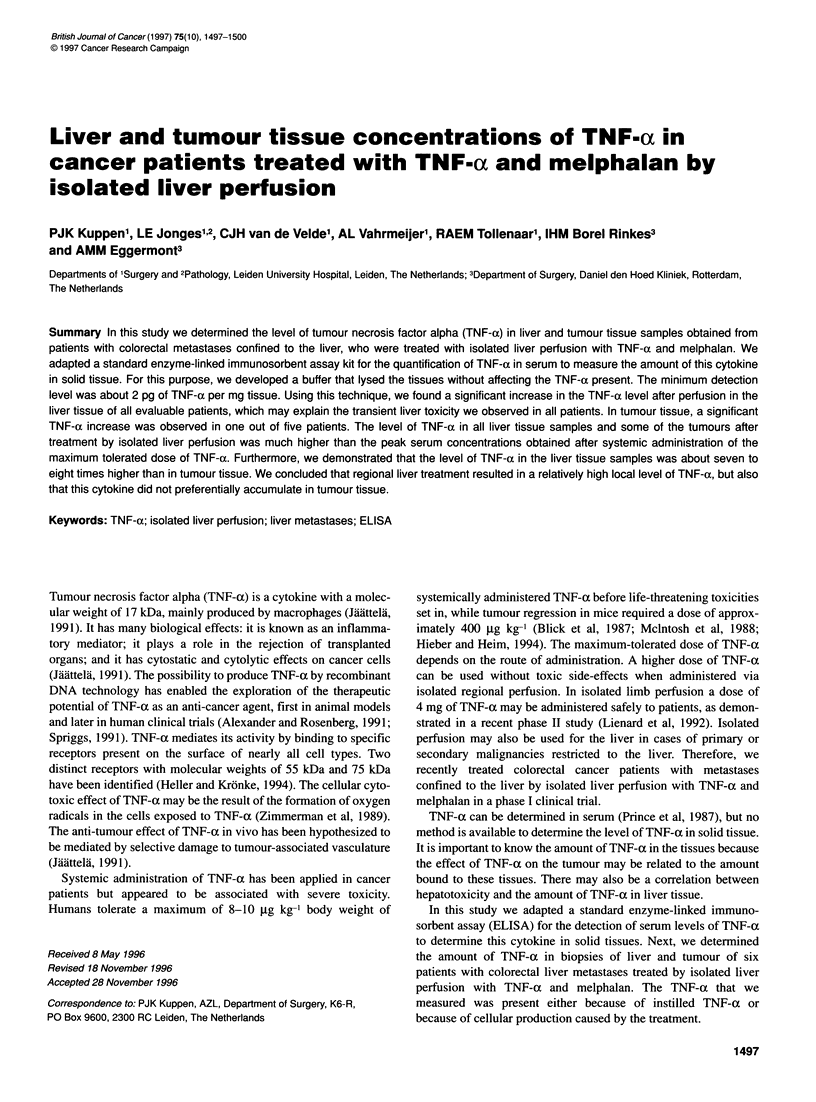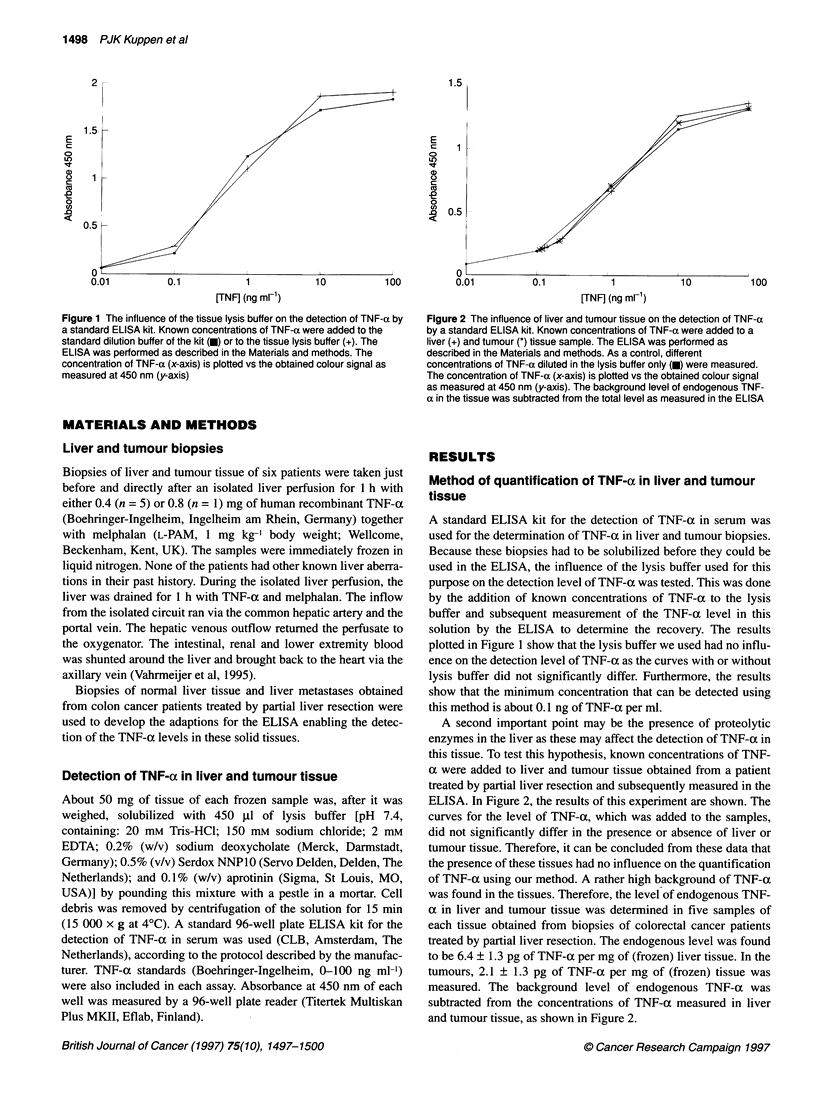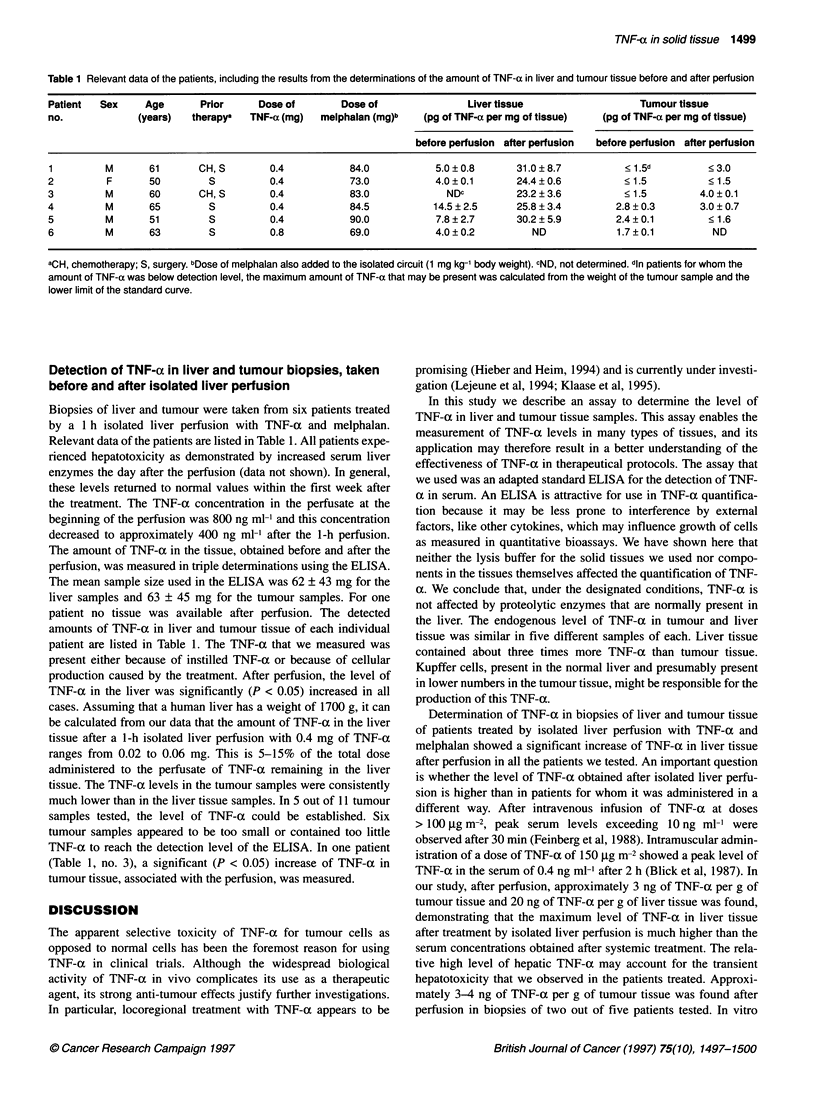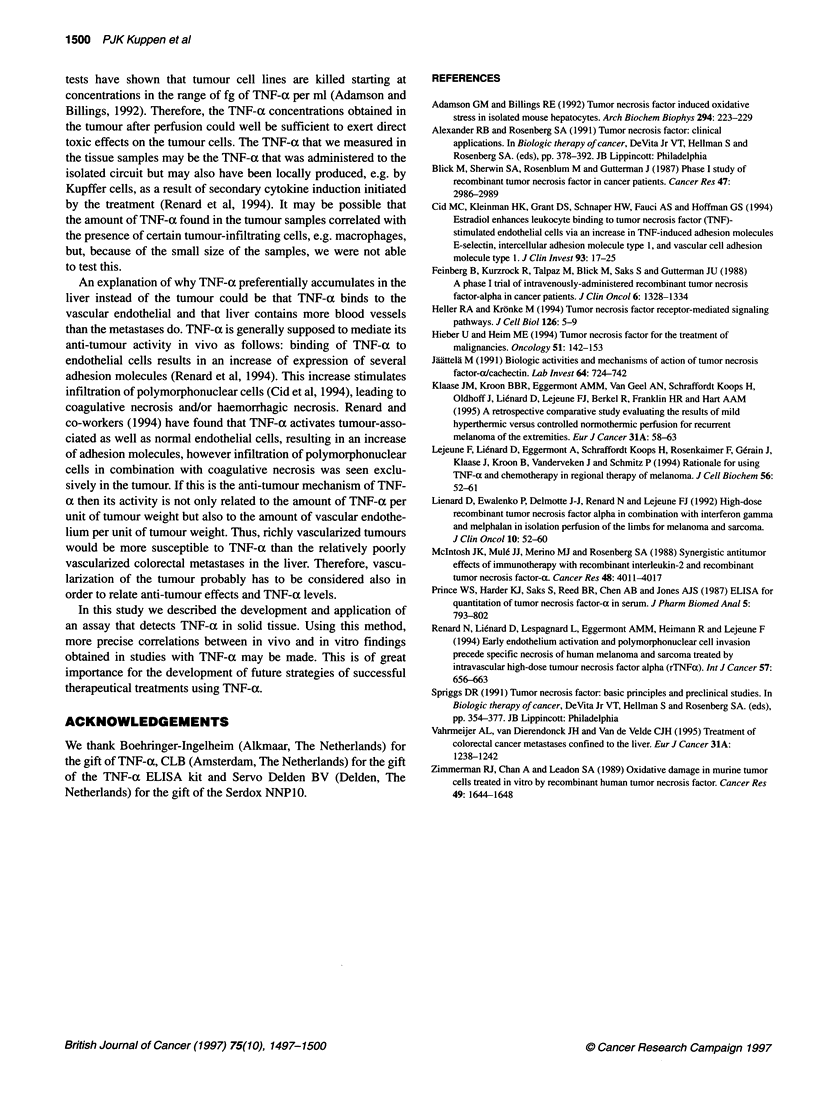# Liver and tumour tissue concentrations of TNF-alpha in cancer patients treated with TNF-alpha and melphalan by isolated liver perfusion.

**DOI:** 10.1038/bjc.1997.255

**Published:** 1997

**Authors:** P. J. Kuppen, L. E. Jonges, C. J. van de Velde, A. L. Vahrmeijer, R. A. Tollenaar, I. H. Borel Rinkes, A. M. Eggermont

**Affiliations:** Department of Surgery, Leiden University Hospital, The Netherlands.

## Abstract

In this study we determined the level of tumour necrosis factor alpha (TNF-alpha) in liver and tumour tissue samples obtained from patients with colorectal metastases confined to the liver, who were treated with isolated liver perfusion with TNF-alpha and melphalan. We adapted a standard enzyme-linked immunosorbent assay kit for the quantification of TNF-alpha in serum to measure the amount of this cytokine in solid tissue. For this purpose, we developed a buffer that lysed the tissues without affecting the TNF-alpha present. The minimum detection level was about 2 pg of TNF-alpha per mg tissue. Using this technique, we found a significant increase in the TNF-alpha level after perfusion in the liver tissue of all evaluable patients, which may explain the transient liver toxicity we observed in all patients. In tumour tissue, a significant TNF-alpha increase was observed in one out of five patients. The level of TNF-alpha in all liver tissue samples and some of the tumours after treatment by isolated liver perfusion was much higher than the peak serum concentrations obtained after systemic administration of the maximum tolerated dose of TNF-alpha. Furthermore, we demonstrated that the level of TNF-alpha in the liver tissue samples was about seven to eight times higher than in tumour tissue. We concluded that regional liver treatment resulted in a relatively high local level of TNF-alpha, but also that this cytokine did not preferentially accumulate in tumour tissue.


British Journal of Cancer (1997) 75(10), 1497–1500
© 1997 Cancer Research Campaign

### Liver and tumour tissue concentrations of TNF-α in cancer patients treated with TNF-α and melphalan by isolated liver perfusion

**PJK Kuppen[1], LE Jonges[1,2], CJH van de Velde[1], AL Vahrmeijer[1], RAEM Tollenaar[1], IHM Borel Rinkes[3] and AMM Eggermont[3]**

Departments of [1]Surgery and [2]Pathology, Leiden University Hospital, Leiden, The Netherlands; [3]Department of Surgery, Daniel den Hoed Kliniek, Rotterdam, The Netherlands

**Summary** In this study we determined the level of tumour necrosis factor alpha (TNF-α) in liver and tumour tissue samples obtained from patients with colorectal metastases confined to the liver, who were treated with isolated liver perfusion with TNF-α and melphalan. We adapted a standard enzyme-linked immunosorbent assay kit for the quantification of TNF-α in serum to measure the amount of this cytokine in solid tissue. For this purpose, we developed a buffer that lysed the tissues without affecting the TNF-α present. The minimum detection level was about 2 pg of TNF-α per mg tissue. Using this technique, we found a significant increase in the TNF-α level after perfusion in the liver tissue of all evaluable patients, which may explain the transient liver toxicity we observed in all patients. In tumour tissue, a significant TNF-α increase was observed in one out of five patients. The level of TNF-α in all liver tissue samples and some of the tumours after treatment by isolated liver perfusion was much higher than the peak serum concentrations obtained after systemic administration of the maximum tolerated dose of TNF-α. Furthermore, we demonstrated that the level of TNF-α in the liver tissue samples was about seven to eight times higher than in tumour tissue. We concluded that regional liver treatment resulted in a relatively high local level of TNF-α, but also that this cytokine did not preferentially accumulate in tumour tissue.

**Keywords:** TNF-α; isolated liver perfusion; liver metastases; ELISA

Tumour necrosis factor alpha (TNF-α) is a cytokine with a molecular weight of 17 kDa, mainly produced by macrophages (Jäättelä, 1991). It has many biological effects: it is known as an inflammatory mediator; it plays a role in the rejection of transplanted organs; and it has cytostatic and cytolytic effects on cancer cells (Jäättelä, 1991). The possibility to produce TNF-α by recombinant DNA technology has enabled the exploration of the therapeutic potential of TNF-α as an anti-cancer agent, first in animal models and later in human clinical trials (Alexander and Rosenberg, 1991; Spriggs, 1991). TNF-α mediates its activity by binding to specific receptors present on the surface of nearly all cell types. Two distinct receptors with molecular weights of 55 kDa and 75 kDa have been identified (Heller and Krönke, 1994). The cellular cytotoxic effect of TNF-α may be the result of the formation of oxygen radicals in the cells exposed to TNF-α (Zimmerman et al, 1989). The anti-tumour effect of TNF-α in vivo has been hypothesized to be mediated by selective damage to tumour-associated vasculature (Jäättelä, 1991).

Systemic administration of TNF-α has been applied in cancer patients but appeared to be associated with severe toxicity. Humans tolerate a maximum of 8–10 μg $kg^{-1}$ body weight of systemically administered TNF-α before life-threatening toxicities set in, while tumour regression in mice required a dose of approximately 400 μg $kg^{-1}$ (Blick et al, 1987; Mclntosh et al, 1988; Hieber and Heim, 1994). The maximum-tolerated dose of TNF-α depends on the route of administration. A higher dose of TNF-α can be used without toxic side-effects when administered via isolated regional perfusion. In isolated limb perfusion a dose of 4 mg of TNF-α may be administered safely to patients, as demonstrated in a recent phase II study (Lienard et al, 1992). Isolated perfusion may also be used for the liver in cases of primary or secondary malignancies restricted to the liver. Therefore, we recently treated colorectal cancer patients with metastases confined to the liver by isolated liver perfusion with TNF-α and melphalan in a phase I clinical trial.

TNF-α can be determined in serum (Prince et al, 1987), but no method is available to determine the level of TNF-α in solid tissue. It is important to know the amount of TNF-α in the tissues because the effect of TNF-α on the tumour may be related to the amount bound to these tissues. There may also be a correlation between hepatotoxicity and the amount of TNF-α in liver tissue.

In this study we adapted a standard enzyme-linked immunosorbent assay (ELISA) for the detection of serum levels of TNF-α to determine this cytokine in solid tissues. Next, we determined the amount of TNF-α in biopsies of liver and tumour of six patients with colorectal liver metastases treated by isolated liver perfusion with TNF-α and melphalan. The TNF-α that we measured was present either because of instilled TNF-α or because of cellular production caused by the treatment.

*Received 8 May 1996*
*Revised 18 November 1996*
*Accepted 28 November 1996*

*Correspondence to:* PJK Kuppen, AZL, Department of Surgery, K6-R, PO Box 9600, 2300 RC Leiden, The Netherlands

**Figure 1** The influence of the tissue lysis buffer on the detection of TNF-α by a standard ELISA kit. Known concentrations of TNF-α were added to the standard dilution buffer of the kit (■) or to the tissue lysis buffer (+). The ELISA was performed as described in the Materials and methods. The concentration of TNF-α (*x*-axis) is plotted vs the obtained colour signal as measured at 450 nm (*y*-axis)

**Figure 2** The influence of liver and tumour tissue on the detection of TNF-α by a standard ELISA kit. Known concentrations of TNF-α were added to a liver (+) and tumour (*) tissue sample. The ELISA was performed as described in the Materials and methods. As a control, different concentrations of TNF-α diluted in the lysis buffer only (■) were measured. The concentration of TNF-α (*x*-axis) is plotted vs the obtained colour signal as measured at 450 nm (*y*-axis). The background level of endogenous TNF-α in the tissue was subtracted from the total level as measured in the ELISA

### MATERIALS AND METHODS

### Liver and tumour biopsies

Biopsies of liver and tumour tissue of six patients were taken just before and directly after an isolated liver perfusion for 1 h with either 0.4 ($n = 5$) or 0.8 ($n = 1$) mg of human recombinant TNF-α (Boehringer-Ingelheim, Ingelheim am Rhein, Germany) together with melphalan (L-PAM, 1 mg kg$^{-1}$ body weight; Wellcome, Beckenham, Kent, UK). The samples were immediately frozen in liquid nitrogen. None of the patients had other known liver aberrations in their past history. During the isolated liver perfusion, the liver was drained for 1 h with TNF-α and melphalan. The inflow from the isolated circuit ran via the common hepatic artery and the portal vein. The hepatic venous outflow returned the perfusate to the oxygenator. The intestinal, renal and lower extremity blood was shunted around the liver and brought back to the heart via the axillary vein (Vahrmeijer et al, 1995).

Biopsies of normal liver tissue and liver metastases obtained from colon cancer patients treated by partial liver resection were used to develop the adaptions for the ELISA enabling the detection of the TNF-α levels in these solid tissues.

### Detection of TNF-α in liver and tumour tissue

About 50 mg of tissue of each frozen sample was, after it was weighed, solubilized with 450 μl of lysis buffer [pH 7.4, containing: 20 mM Tris-HCl; 150 mM sodium chloride; 2 mM EDTA; 0.2% (w/v) sodium deoxycholate (Merck, Darmstadt, Germany); 0.5% (v/v) Serdox NNP10 (Servo Delden, Delden, The Netherlands); and 0.1% (w/v) aprotinin (Sigma, St Louis, MO, USA)] by pounding this mixture with a pestle in a mortar. Cell debris was removed by centrifugation of the solution for 15 min (15 000 × **g** at 4°C). A standard 96-well plate ELISA kit for the detection of TNF-α in serum was used (CLB, Amsterdam, The Netherlands), according to the protocol described by the manufacturer. TNF-α standards (Boehringer-Ingelheim, 0–100 ng ml$^{-1}$) were also included in each assay. Absorbance at 450 nm of each well was measured by a 96-well plate reader (Titertek Multiskan Plus MKII, Eflab, Finland).

### RESULTS

### Method of quantification of TNF-α in liver and tumour tissue

A standard ELISA kit for the detection of TNF-α in serum was used for the determination of TNF-α in liver and tumour biopsies. Because these biopsies had to be solubilized before they could be used in the ELISA, the influence of the lysis buffer used for this purpose on the detection level of TNF-α was tested. This was done by the addition of known concentrations of TNF-α to the lysis buffer and subsequent measurement of the TNF-α level in this solution by the ELISA to determine the recovery. The results plotted in Figure 1 show that the lysis buffer we used had no influence on the detection level of TNF-α as the curves with or without lysis buffer did not significantly differ. Furthermore, the results show that the minimum concentration that can be detected using this method is about 0.1 ng of TNF-α per ml.

A second important point may be the presence of proteolytic enzymes in the liver as these may affect the detection of TNF-α in this tissue. To test this hypothesis, known concentrations of TNF-α were added to liver and tumour tissue obtained from a patient treated by partial liver resection and subsequently measured in the ELISA. In Figure 2, the results of this experiment are shown. The curves for the level of TNF-α, which was added to the samples, did not significantly differ in the presence or absence of liver or tumour tissue. Therefore, it can be concluded from these data that the presence of these tissues had no influence on the quantification of TNF-α using our method. A rather high background of TNF-α was found in the tissues. Therefore, the level of endogenous TNF-α in liver and tumour tissue was determined in five samples of each tissue obtained from biopsies of colorectal cancer patients treated by partial liver resection. The endogenous level was found to be 6.4 ± 1.3 pg of TNF-α per mg of (frozen) liver tissue. In the tumours, 2.1 ± 1.3 pg of TNF-α per mg of (frozen) tissue was measured. The background level of endogenous TNF-α was subtracted from the concentrations of TNF-α measured in liver and tumour tissue, as shown in Figure 2.

© Cancer Research Campaign 1997

**Table 1** Relevant data of the patients, including the results from the determinations of the amount of TNF-α in liver and tumour tissue before and after perfusion

| Patient no. | Sex | Age (years) | Prior therapy[a] | Dose of TNF-α (mg) | Dose of melphalan (mg)[b] | Liver tissue (pg of TNF-α per mg of tissue) | | Tumour tissue (pg of TNF-α per mg of tissue) | |
|---|---|---|---|---|---|---|---|---|---|
| | | | | | | before perfusion | after perfusion | before perfusion | after perfusion |
| 1 | M | 61 | CH, S | 0.4 | 84.0 | 5.0 ± 0.8 | 31.0 ± 8.7 | ≤ 1.5[d] | ≤ 3.0 |
| 2 | F | 50 | S | 0.4 | 73.0 | 4.0 ± 0.1 | 24.4 ± 0.6 | ≤ 1.5 | ≤ 1.5 |
| 3 | M | 60 | CH, S | 0.4 | 83.0 | ND[c] | 23.2 ± 3.6 | ≤ 1.5 | 4.0 ± 0.1 |
| 4 | M | 65 | S | 0.4 | 84.5 | 14.5 ± 2.5 | 25.8 ± 3.4 | 2.8 ± 0.3 | 3.0 ± 0.7 |
| 5 | M | 51 | S | 0.4 | 90.0 | 7.8 ± 2.7 | 30.2 ± 5.9 | 2.4 ± 0.1 | ≤ 1.6 |
| 6 | M | 63 | S | 0.8 | 69.0 | 4.0 ± 0.2 | ND | 1.7 ± 0.1 | ND |

[a]CH, chemotherapy; S, surgery. [b]Dose of melphalan also added to the isolated circuit (1 mg kg$^{-1}$ body weight). [c]ND, not determined. [d]In patients for whom the amount of TNF-α was below detection level, the maximum amount of TNF-α that may be present was calculated from the weight of the tumour sample and the lower limit of the standard curve.

### Detection of TNF-α in liver and tumour biopsies, taken before and after isolated liver perfusion

Biopsies of liver and tumour were taken from six patients treated by a 1 h isolated liver perfusion with TNF-α and melphalan. Relevant data of the patients are listed in Table 1. All patients experienced hepatotoxicity as demonstrated by increased serum liver enzymes the day after the perfusion (data not shown). In general, these levels returned to normal values within the first week after the treatment. The TNF-α concentration in the perfusate at the beginning of the perfusion was 800 ng ml$^{-1}$ and this concentration decreased to approximately 400 ng ml$^{-1}$ after the 1-h perfusion. The amount of TNF-α in the tissue, obtained before and after the perfusion, was measured in triple determinations using the ELISA. The mean sample size used in the ELISA was 62 ± 43 mg for the liver samples and 63 ± 45 mg for the tumour samples. For one patient no tissue was available after perfusion. The detected amounts of TNF-α in liver and tumour tissue of each individual patient are listed in Table 1. The TNF-α that we measured was present either because of instilled TNF-α or because of cellular production caused by the treatment. After perfusion, the level of TNF-α in the liver was significantly ($P < 0.05$) increased in all cases. Assuming that a human liver has a weight of 1700 g, it can be calculated from our data that the amount of TNF-α in the liver tissue after a 1-h isolated liver perfusion with 0.4 mg of TNF-α ranges from 0.02 to 0.06 mg. This is 5–15% of the total dose administered to the perfusate of TNF-α remaining in the liver tissue. The TNF-α levels in the tumour samples were consistently much lower than in the liver tissue samples. In 5 out of 11 tumour samples tested, the level of TNF-α could be established. Six tumour samples appeared to be too small or contained too little TNF-α to reach the detection level of the ELISA. In one patient (Table 1, no. 3), a significant ($P < 0.05$) increase of TNF-α in tumour tissue, associated with the perfusion, was measured.

### DISCUSSION

The apparent selective toxicity of TNF-α for tumour cells as opposed to normal cells has been the foremost reason for using TNF-α in clinical trials. Although the widespread biological activity of TNF-α in vivo complicates its use as a therapeutic agent, its strong anti-tumour effects justify further investigations. In particular, locoregional treatment with TNF-α appears to be promising (Hieber and Heim, 1994) and is currently under investigation (Lejeune et al, 1994; Klaase et al, 1995).

In this study we describe an assay to determine the level of TNF-α in liver and tumour tissue samples. This assay enables the measurement of TNF-α levels in many types of tissues, and its application may therefore result in a better understanding of the effectiveness of TNF-α in therapeutical protocols. The assay that we used was an adapted standard ELISA for the detection of TNF-α in serum. An ELISA is attractive for use in TNF-α quantification because it may be less prone to interference by external factors, like other cytokines, which may influence growth of cells as measured in quantitative bioassays. We have shown here that neither the lysis buffer for the solid tissues we used nor components in the tissues themselves affected the quantification of TNF-α. We conclude that, under the designated conditions, TNF-α is not affected by proteolytic enzymes that are normally present in the liver. The endogenous level of TNF-α in tumour and liver tissue was similar in five different samples of each. Liver tissue contained about three times more TNF-α than tumour tissue. Kupffer cells, present in the normal liver and presumably present in lower numbers in the tumour tissue, might be responsible for the production of this TNF-α.

Determination of TNF-α in biopsies of liver and tumour tissue of patients treated by isolated liver perfusion with TNF-α and melphalan showed a significant increase of TNF-α in liver tissue after perfusion in all the patients we tested. An important question is whether the level of TNF-α obtained after isolated liver perfusion is higher than in patients for whom it was administered in a different way. After intravenous infusion of TNF-α at doses > 100 μg m$^{-2}$, peak serum levels exceeding 10 ng ml$^{-1}$ were observed after 30 min (Feinberg et al, 1988). Intramuscular administration of a dose of TNF-α of 150 μg m$^{-2}$ showed a peak level of TNF-α in the serum of 0.4 ng ml$^{-1}$ after 2 h (Blick et al, 1987). In our study, after perfusion, approximately 3 ng of TNF-α per g of tumour tissue and 20 ng of TNF-α per g of liver tissue was found, demonstrating that the maximum level of TNF-α in liver tissue after treatment by isolated liver perfusion is much higher than the serum concentrations obtained after systemic treatment. The relative high level of hepatic TNF-α may account for the transient hepatotoxicity that we observed in the patients treated. Approximately 3–4 ng of TNF-α per g of tumour tissue was found after perfusion in biopsies of two out of five patients tested. In vitro

© Cancer Research Campaign 1997

tests have shown that tumour cell lines are killed starting at concentrations in the range of fg of TNF-α per ml (Adamson and Billings, 1992). Therefore, the TNF-α concentrations obtained in the tumour after perfusion could well be sufficient to exert direct toxic effects on the tumour cells. The TNF-α that we measured in the tissue samples may be the TNF-α that was administered to the isolated circuit but may also have been locally produced, e.g. by Kupffer cells, as a result of secondary cytokine induction initiated by the treatment (Renard et al, 1994). It may be possible that the amount of TNF-α found in the tumour samples correlated with the presence of certain tumour-infiltrating cells, e.g. macrophages, but, because of the small size of the samples, we were not able to test this.

An explanation of why TNF-α preferentially accumulates in the liver instead of the tumour could be that TNF-α binds to the vascular endothelial and that liver contains more blood vessels than the metastases do. TNF-α is generally supposed to mediate its anti-tumour activity in vivo as follows: binding of TNF-α to endothelial cells results in an increase of expression of several adhesion molecules (Renard et al, 1994). This increase stimulates infiltration of polymorphonuclear cells (Cid et al, 1994), leading to coagulative necrosis and/or haemorrhagic necrosis. Renard and co-workers (1994) have found that TNF-α activates tumour-associated as well as normal endothelial cells, resulting in an increase of adhesion molecules, however infiltration of polymorphonuclear cells in combination with coagulative necrosis was seen exclusively in the tumour. If this is the anti-tumour mechanism of TNF-α then its activity is not only related to the amount of TNF-α per unit of tumour weight but also to the amount of vascular endothelium per unit of tumour weight. Thus, richly vascularized tumours would be more susceptible to TNF-α than the relatively poorly vascularized colorectal metastases in the liver. Therefore, vascularization of the tumour probably has to be considered also in order to relate anti-tumour effects and TNF-α levels.

In this study we described the development and application of an assay that detects TNF-α in solid tissue. Using this method, more precise correlations between in vivo and in vitro findings obtained in studies with TNF-α may be made. This is of great importance for the development of future strategies of successful therapeutical treatments using TNF-α.

### ACKNOWLEDGEMENTS

We thank Boehringer-Ingelheim (Alkmaar, The Netherlands) for the gift of TNF-α, CLB (Amsterdam, The Netherlands) for the gift of the TNF-α ELISA kit and Servo Delden BV (Delden, The Netherlands) for the gift of the Serdox NNP10.

### REFERENCES

Adamson GM and Billings RE (1992) Tumor necrosis factor induced oxidative stress in isolated mouse hepatocytes. *Arch Biochem Biophys* **294**: 223–229

Alexander RB and Rosenberg SA (1991) Tumor necrosis factor: clinical applications. In *Biologic therapy of cancer*, DeVita Jr VT, Hellman S and Rosenberg SA. (eds), pp. 378–392. JB Lippincott: Philadelphia

Blick M, Sherwin SA, Rosenblum M and Gutterman J (1987) Phase I study of recombinant tumor necrosis factor in cancer patients. *Cancer Res* **47**: 2986–2989

Cid MC, Kleinman HK, Grant DS, Schnaper HW, Fauci AS and Hoffman GS (1994) Estradiol enhances leukocyte binding to tumor necrosis factor (TNF)-stimulated endothelial cells via an increase in TNF-induced adhesion molecules E-selectin, intercellular adhesion molecule type 1, and vascular cell adhesion molecule type 1. *J Clin Invest* **93**: 17–25

Feinberg B, Kurzrock R, Talpaz M, Blick M, Saks S and Gutterman JU (1988) A phase I trial of intravenously-administered recombinant tumor necrosis factor-alpha in cancer patients. *J Clin Oncol* **6**: 1328–1334

Heller RA and Krönke M (1994) Tumor necrosis factor receptor-mediated signaling pathways. *J Cell Biol* **126**: 5–9

Hieber U and Heim ME (1994) Tumor necrosis factor for the treatment of malignancies. *Oncology* **51**: 142–153

Jäättelä M (1991) Biologic activities and mechanisms of action of tumor necrosis factor-α/cachectin. *Lab Invest* **64**: 724–742

Klaase JM, Kroon BBR, Eggermont AMM, Van Geel AN, Schraffordt Koops H, Oldhoff J, Liénard D, Lejeune FJ, Berkel R, Franklin HR and Hart AAM (1995) A retrospective comparative study evaluating the results of mild hyperthermic versus controlled normothermic perfusion for recurrent melanoma of the extremities. *Eur J Cancer* **31A**: 58–63

Lejeune F, Liénard D, Eggermont A, Schraffordt Koops H, Rosenkaimer F, Gérain J, Klaase J, Kroon B, Vanderveken J and Schmitz P (1994) Rationale for using TNF-α and chemotherapy in regional therapy of melanoma. *J Cell Biochem* **56**: 52–61

Lienard D, Ewalenko P, Delmotte J-J, Renard N and Lejeune FJ (1992) High-dose recombinant tumor necrosis factor alpha in combination with interferon gamma and melphalan in isolation perfusion of the limbs for melanoma and sarcoma. *J Clin Oncol* **10**: 52–60

McIntosh JK, Mulé JJ, Merino MJ and Rosenberg SA (1988) Synergistic antitumor effects of immunotherapy with recombinant interleukin-2 and recombinant tumor necrosis factor-α. *Cancer Res* **48**: 4011–4017

Prince WS, Harder KJ, Saks S, Reed BR, Chen AB and Jones AJS (1987) ELISA for quantitation of tumor necrosis factor-α in serum. *J Pharm Biomed Anal* **5**: 793–802

Renard N, Liénard D, Lespagnard L, Eggermont AMM, Heimann R and Lejeune F (1994) Early endothelium activation and polymorphonuclear cell invasion precede specific necrosis of human melanoma and sarcoma treated by intravascular high-dose tumour necrosis factor alpha (rTNFα). *Int J Cancer* **57**: 656–663

Spriggs DR (1991) Tumor necrosis factor: basic principles and preclinical studies. In *Biologic therapy of cancer*, DeVita Jr VT, Hellman S and Rosenberg SA. (eds), pp. 354–377. JB Lippincott: Philadelphia

Vahrmeijer AL, van Dierendonck JH and Van de Velde CJH (1995) Treatment of colorectal cancer metastases confined to the liver. *Eur J Cancer* **31A**: 1238–1242

Zimmerman RJ, Chan A and Leadon SA (1989) Oxidative damage in murine tumor cells treated in vitro by recombinant human tumor necrosis factor. *Cancer Res* **49**: 1644–1648

© Cancer Research Campaign 1997